# Ferroptosis-related genes are involved in asthma and regulate the immune microenvironment

**DOI:** 10.3389/fphar.2023.1087557

**Published:** 2023-02-10

**Authors:** Haixia Wang, Yuanmin Jia, Junlian Gu, Ou Chen, Shouwei Yue

**Affiliations:** ^1^ School of Nursing and Rehabilitation, Cheeloo College of Medicine, Shandong University, Jinan, Shandong, China; ^2^ Rehabilitation Center, Qilu Hospital of Shandong University, Jinan, Shandong, China

**Keywords:** asthma, ferroptosis, immune infiltration, co-expression network analysis, tregs (regulatory T-cell)

## Abstract

**Background:** Asthma was a chronic inflammatory illness driven by complicated genetic regulation and environmental exposure. The complex pathophysiology of asthma has not been fully understood. Ferroptosis was involved in inflammation and infection. However, the effect of ferroptosis on asthma was still unclear. The study was designed to identify ferroptosis-related genes in asthma, providing potential therapeutic targets.

**Methods:** We conducted a comprehensive analysis combined with WGCNA, PPI, GO, KEGG, and CIBERSORT methods to identify ferroptosis-related genes that were associated with asthma and regulated the immune microenvironment in GSE147878 from the GEO. The results of this study were validated in GSE143303 and GSE27066, and the hub genes related to ferroptosis were further verified by immunofluorescence and RT-qPCR in the OVA asthma model.

**Results:** 60 asthmatics and 13 healthy controls were extracted for WGCNA. We found that genes in the black module (r = −0.47, *p* < 0.05) and magenta module (r = 0.51, *p* < 0.05) were associated with asthma. CAMKK2 and CISD1 were discovered to be ferroptosis-related hub genes in the black and magenta module, separately. We found that CAMKK2 and CISD1 were mainly involved in the CAMKK-AMPK signaling cascade, the adipocytokine signaling pathway, the metal cluster binding, iron-sulfur cluster binding, and 2 iron, 2 sulfur cluster binding in the enrichment analysis, which was strongly correlated with the development of ferroptosis. We found more infiltration of M2 macrophages and less Tregs infiltration in the asthma group compared to healthy controls. In addition, the expression levels of CISD1 and Tregs were negatively correlated. Through validation, we found that CAMKK2 and CISD1 expression were upregulated in the asthma group compared to the control group and would inhibit the occurrence of ferroptosis.

**Conclusion:** CAMKK2 and CISD1 might inhibit ferroptosis and specifically regulate asthma. Moreover, CISD1 might be tied to the immunological microenvironment. Our results could be useful to provide potential immunotherapy targets and prognostic markers for asthma.

## 1 Introduction

Asthma was recognized as one of the most common chronic diseases driven by interactions between genetic regulation and environmental exposure (Vos et al., 2012; Kaur and Chupp, 2019). Current asthma therapy was based mainly on inhaled corticosteroids and bronchodilators to suppress symptoms, and the symptoms of about 5%–10% of patients with asthma were not adequately controlled (Papi et al., 2018). Little success has been achieved in developing drugs that target the underlying mechanisms of asthma, which implied that understanding the genetic basis of asthma might unravel many disease-causing mechanisms (El-Husseini et al., 2020).

Ferroptosis after being first reported by Dixon et al., in 2012 has been recognized as a potential therapeutic target in many diseases (Hassannia et al., 2019; Qiu et al., 2020; Ma et al., 2021). Ferroptosis was a unique intracellular iron-dependent form of non-apoptotic cell death that occurred through excessive peroxidation of polyunsaturated fatty acids (Dixon et al., 2012). Recent studies have also indicated that ferroptosis played a critical role in the pathogenesis of lung diseases (Xu et al., 2021). Some researchers have also initially explored the role that ferroptosis acted in asthma. Wu et al. (2020) found that the induction of ferroptosis alleviated allergic inflammation in the OVA asthma model. In contrast, Zeng et al. (2022) showed that ferroptosis might occur in airway epithelial cells, and the inhibitors of ferroptosis could reduce levels of IL4, IL5, and IL13 in the HDM asthma model. Most of the currently published research on ferroptosis and asthma has focused on animal models of asthma, and only Nagasaki et al. have explored the potential mechanisms of ferroptosis in asthmatics (Bao et al., 2022; Nagasaki et al., 2022). Furthermore, the results of the relationship between ferroptosis and asthma were controversial.

FerrDb was the first database of experimentally validated ferroptosis regulators and markers and ferroptosis-disease associations (Zhou and Bao, 2020). Several researchers took advantage of this database in recent years to select ferroptosis-related genes in various diseases. In addition, Weighted Gene Co-expression analysis (WGCNA) was used as a data exploratory tool or as a gene screening method and aimed to provide potential disease-related molecular targets (Langfelder and Horvath, 2008). Therefore, we first screened for asthma-related specific modules using the WGCNA approach, and then further filtered for ferroptosis genes associated with asthma in combination with the FerrDb database. In this way, we want to clarify the molecular process by which genes associated with ferroptosis were engaged in asthma, which might help us find molecular targets that might aid in the early diagnosis of asthma and improve patient treatment.

## 2 Methods

### 2.1 Data download and processing

GSE147878 was downloaded from the Gene Expression Omnibus (GEO) database (https://www.ncbi.nlm.nih.gov/geo). It was part of the U-BIOPRED cohort and the Australian Newcastle severe asthma cohort including endobronchial biopsies of 60 asthmatics (including 18 mild/moderate asthma and 42 severe asthma) and 13 healthy controls. The criteria for diagnosis of asthma were agreed upon at the U-BIOPRED consensus meeting. (1) Airflow reversibility: Participants with asthma had an increase in forced expiratory volume in 1 s (FEV_1_) >12% predicted or 200 mL after inhaling 400 µg salbutamol; (2) Airway hyperresponsiveness: methacholine provocative concentration caused a 20% fall in FEV_1_ <8 mgmL^−1^, or diurnal peak expiratory flow (PEF) amplitude >8% of mean; (3) Participants with asthma had a decrease in FEV_1_ of 12% predicted or 200 mL within 4 weeks after tapering maintenance treatment. Healthy controls had no history of asthma or wheezing, no other chronic respiratory disease, and their pre-bronchodilator FEV1 was ≥80% pred (Shaw et al., 2015).

First, we used the GEOquery package of the R software (version 4.1.0) to download the GSE147878 from the GEO database. Second, the raw gene expression data were normalized by the robust multi-array average (RMA) method by the R Bioconductor package affy (Bolstad et al., 2003). Third, we used the annotation information to map gene IDs to microarray probes. Probes matching more than one gene were excluded and the mean expression value of genes measured by multiple probes was calculated. Four, the top 5,000 genes were screened using the median absolute deviation (MAD) value (Zhao et al., 2021). Finally, we imported data on clinical characteristics including age, atopy, oral corticosteroid (OCS), gender, prednisone, and smokers. The false discovery rate (FDR) technique modified the *p*-value.

### 2.2 Network construction and consensus module detection

WGCNA was used to separate genes into various clusters and further explore the connection between co-expression modules and clinical characteristics. All co-expression networks were created using the Bioconductor WGCNA package (Langfelder and Horvath, 2008). (1) Samples were clustered and looked for outliers using the hclust function. (2) The pickSoftThreshold function of WGCNA, which computed the scale-free topology fit index for a set of candidate powers ranging from 1 to 20, was used to determine the soft-thresholding power in the building of each module. If the index value for the reference dataset was more than 0.85, the appropriate power was found. (3) Co-expression modules were discovered using one-step network construction, with a minimum gene number of 50.

### 2.3 Relating modules to external clinical traits

The gene significance (GS) and module membership (MM) were used to identify the gene of high group significance and module membership in the modules. The significant module for asthma was identified if: |GS| ≥ 0.5 and |MM| ≥ 0.5, and hub genes were visualized in the Protein-Protein Interaction (PPI) network based on the STRING database. The relationship between MM and GS in the module was statistically significant (*p* < 0.05).

### 2.4 Identification of hub genes related to ferroptosis

FerrDb V2 was the first database of experimentally validated ferroptosis regulators and markers and ferroptosis-disease associations (Zhou and Bao, 2020). The ferroptosis-related genes were downloaded to the FerrDb database (http://www.zhounan.org/ferrdb/), including the driver, suppressor, and marker genes. These genes were compared to asthma-related genes generated from WGCNA. The overlapping genes were described by the Venn diagram.

### 2.5 Enrichment analysis

To further visualize the biological function of key genes related to ferroptosis in the key module, Gene Ontology (GO) and Kyoto Encyclopedia of Genes and Genomes (KEGG) enrichment analyses were identified in the Cytoscape plug-in ClueGo. The *p*-value of less than 0.05 was identified as a significant term.

### 2.6 Construction of ceRNA network

The RNA Interactome Database (RNAInter) was a comprehensive resource for RNA interactome data obtained from the literature and other databases, containing over 41 million RNA-associated interactions (Lin et al., 2020). CDGSH iron sulfur domain 1 (CISD1) and Calcium/calmodulin-dependent protein kinase 2 (CAMKK2) were entered into the RNAInter website respectively to get miRNA with a confidence score >0.5 related to the CISD1 and CAMKK2. We then entered these miRNAs into RNAInter to search for miRNA-associated lncRNAs with a confidence score >0.5. The result of ceRNA was visualized by a Sankey diagram in the river plot package.

### 2.7 Immune infiltrating

CIBERSORT was a deconvolution method for accurately quantifying cell fractions from gene expression profiles (Newman et al., 2015; Chen et al., 2018). LM22 gene signature files provided by CIBERSORT were used to estimate the abundances of immunocytes accompanying 1,000 permutations. In addition, the Wilcoxon test was applied to establish the differentially infiltrated immune cells in asthma compared to healthy control. The correlation between hub genes and immune infiltrating cells was analyzed by Spearman’s rank correlation analysis. The results were visualized by the lollipop chart using the “ggplot2” and “ggpubr” packages.

### 2.8 Validation in the GEO dataset

Considering that the asthma group in this study was including patients with mild to moderate asthma and severe asthma, we validated the results of this study in mild to moderate asthma and severe asthma separately to avoid our findings being the main effect of a particular phenotype of mild to moderate asthma or severe asthma. The characteristics of participants in the GSE147878 were shown in Table S1. Furthermore, to validate the robustness of the results, we validated the results in the GSE143303 and GSE27066 datasets. The GSE143303 included endobronchial biopsies of adults with severe asthma (*n* = 47), and healthy controls (*n* = 13). The characteristics of participants in the GSE143303 were shown in Table S2. In addition, we also validated our results in animal models of asthma in the GSE27066. The mice model of asthma was constructed as follows (*n* = 4 per group): Mice were immunized with 3 intraperitoneal injections of 50 μg OVA (Sigma-Aldrich) in 0.1 mL PBS on days 1, 4, and 7. Starting on day 12, mice have challenged with 20 μg OVA in 30 μL PBS weekly for 9 weeks; control mice received intraperitoneal injections and were challenged with PBS at the same time (Yu et al., 2011).

### 2.9 Validation in the OVA models

#### 2.9.1 Animals and treatments

We have also constructed OVA models to validate the bioinformatic results. The OVA model was constructed based on the study by Shen et al. (2003). C57BL/6J male mice were purchased from Vital River Laboratories (Beijing, China). Mice were allowed tap water and rodent chow and were maintained at 22°C with a 12 h light-12 h dark cycle. All mice were acclimatized for 1 week before experimentation. The C57BL/6 mice were randomly divided into 2 groups (*n* = 4 per group). The OVA group was immunized intraperitoneally with OVA and challenged with inhaled OVA. Briefly, mice were injected with 100 µL of 20 µg chicken OVA (Sigma,United States) emulsified in Imject alum (Pierce, United States) on days 0 and 14, and subsequently challenged for 40 min with an aerosol generated by ultrasonic nebulization of 2% OVA in saline from 24 to 41 days. The control group was replaced with saline in both the sensitization and excitation phases. All experimental procedures used in this study were approved and conducted according to the guidelines of the laboratory Animal Management Committee of Shandong University.

#### 2.9.2 Real-time quantitative PCR

The gene expression was tested by real-time quantitative PCR (RT-qPCR). Briefly, TRIzol reagent (Cwbio, Jiangsu, China) was used for total RNA extraction of lung tissues. RNA was reverse transcribed using a HiFiScript cDNA Synthesis Kit (Cwbio), and cDNA was synthesized by reverse transcription using the HiFiScript cDNA Synthesis Kit (Cwbio). RT-qPCR was carried out using an UltraSYBR Mixture (Cwbio) and real-time PCR detection equipment (Bio-Rad, Hercules, CA, United States). Primers used as follows: mouse CAMKK2 (Forward: GGA​GGA​CGA​GAA​CTG​CAC​AC, Reverse: TTC​GCT​GCC​TTG​CTT​CGT​GA) mouse CISD1(Forward: GCT​GTG​CGA​GTT​GA-GTG​GAT, Reverse: TGG​TGC​GAT​T-CTC​TTT​AGC​GTA), and mouse GAPDH (Forward: GGC​CCC​TCT​GGA​AAG​CTG​TGG, Reverse: CCC​GGC​ATC​GAA​GGT-GGA​AGA) were purchased from Sangon Biotech (Shanghai, China). The *t*-test (two-tailed) was used to compare the expression differences between the groups in GraphPad Prism 9. *p* < 0.05 was considered statistically significant.

#### 2.9.3 Immunofluorescent staining

For immunofluorescent (IF) staining, frozen myocardial tissues were fixed with 4% paraformaldehyde for 20 min Primary antibodies, anti-CISD1 antibody at dilution of 1:25, and anti-CAMKK2 antibody at dilution of 1:200 (Proteintech, Chicago, United States) were used, respectively. The secondary antibody was coralite 594-conjugated goat anti-rabbit Ig G (H + L) (1:200, Proteintech, Chicago, United States). DAPI was applied to stain nuclei (blue). All the above staining was conducted according to the manufacturer’s instructions. Staining results were observed and photographed by the fluorescence microscope (Nikon, Tokyo, Japan) and calculated by ImageJ software.

## 3 Results

### 3.1 Co-expression network construction

**FIGURE 1:**
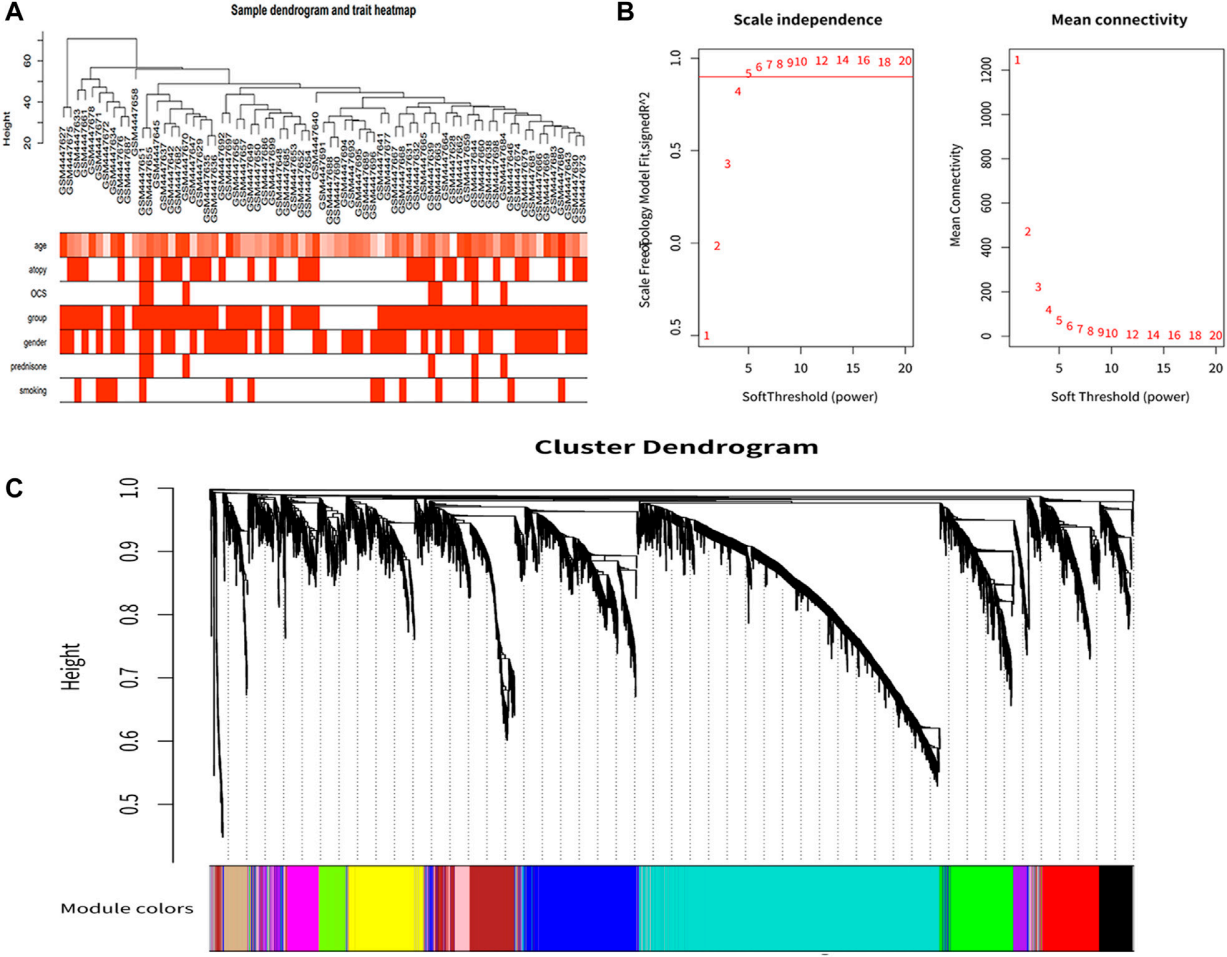
Co-expression network construction. **(A)** Sample clustering dendrogram based on Euclidean distance. **(B)** Analysis of network topology for various soft-thresholding powers. With a scale-free topological criterion >0.9, 5 was chosen as the fittest power value. **(C)** Clustering dendrogram of genes, with dissimilarity based on the topological overlap, together with assigned module colors.

After clustering all samples, we did not discover any outliers in Figure 1A. The soft thresholding power β was set at 5 when the scale independence reached 0.9 in Figure 1B. Finally, 12 gene co-expression modules were constructed after using the one-step network construction function of the WGCNA R package (Figure 1C).

### 3.2 Identification of the clinically significant module and hub genes

**FIGURE 2:**
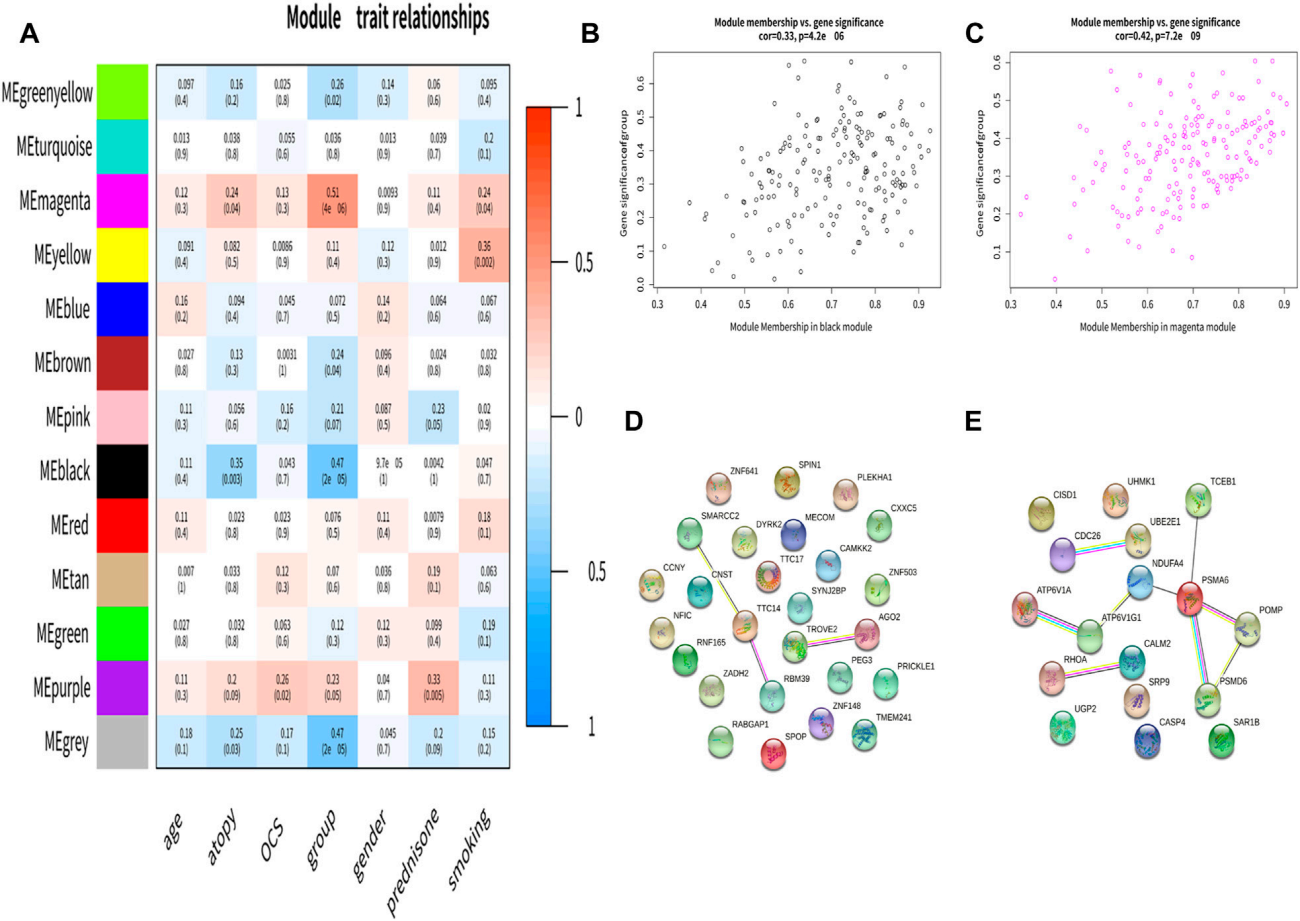
Identification of the clinically significant module and hub genes. **(A)** Module-trait associations. Each row corresponds to a module eigengene, column to a clinical trait. **(B,C)** A scatterplot of Gene Significance (GS) for asthma vs. Module Membership (MM) in the black and magenta module. **(D,E)** The PPI network in the black and magenta modules.

The correlation between module eigengene and clinical features was used to identify module-trait associations (Figure 2A). The black module was shown to be adversely associated with asthma, with correlations of 0.47 (*p* < 0.05). This indicated that genes in the black module were predominantly downregulated in asthmatics. The magenta module was recognized as the positive module with a correlation of 0.51(*p*< 0.05). Figures 2B, C, showed that the black, magenta modules had the GS-MM correlation (*p* < 0.05). Finally, we found 26 and 17 key genes in the black and magenta modules (Figures 2D, E).

### 3.3 Identification of differentially expressed genes related to ferroptosis

**FIGURE 3:**
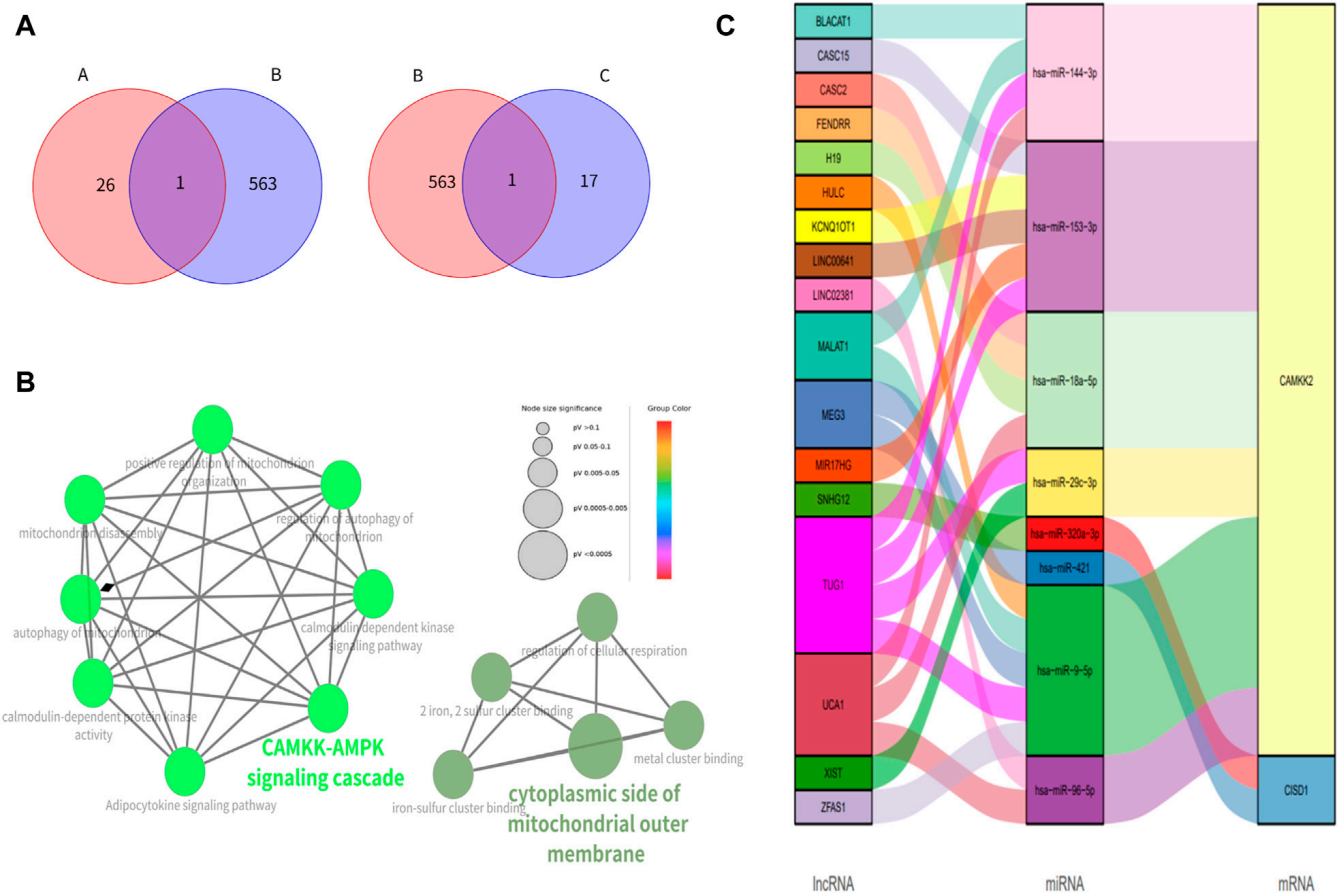
Identification of genes related to ferroptosis, enrichment analysis of ferroptosis-associated genes and construction of ceRNA networks **(A)** The Venn figure for intersected genes in FerrBD and different modules (A: black module; B: FerrBD; C: magenta module) **(B)** Functional enrichment of CISD1 AND CAMKK2. The signal pathways were discovered by enrichment analysis into groups based on functional connection, the same group was colored the same color, and the labels of each group of the most essential terms were color-coded. **(C)** The lncRNA-miRNA-mRNA ceRNA network of CISD1 and CAMKK2.

The ferroptosis-related genes including 369 drivers, 348 suppressors, 11 markers, and 116 unclassified were downloaded from the FerrDb. After removing duplicate genes, we obtained 563 genes related to ferroptosis. After overlapping the hub genes in the black module with genes in the FerrDb database, we found that CAMKK2 was associated with ferroptosis in the black module. And only CISD1 in the magenta module was associated with ferroptosis (Figure 3A). We further identified CAMKK2 and CISD1 as suppressors of ferroptosis by FerrDb.

### 3.4 Enrichment analysis

GO analysis showed that CISD1 was mainly located in the cytoplasmic side of the mitochondrial outer membrane, meanwhile, it was involved in the metal cluster binding, iron-sulfur cluster binding, 2 iron, 2 sulfur cluster binding, and regulation of cellular respiration. We found that CAMKK2 was associated with CAMKK-AMPK signaling cascade, calmodulin-dependent kinase signaling pathway, autophagy of mitochondrion, positive regulation of mitochondrion organization, mitochondrion disascembly and regulation of autophagy of mitochondrion in the GO analysis. KEGG pathway analysis showed that CAMKK2 was mainly involved in the Adipocytokine signaling pathway (Figure 3B).

### 3.5 Construction of ceRNA network

We discovered that lncRNA such as BLACAT1, CASC15, and CASC2 acted as the miRNA sponge and thus cause the upregulation of CISD1 and CAMKK2 (Figure 3C).

### 3.6 Immune infiltrating

**FIGURE 4:**
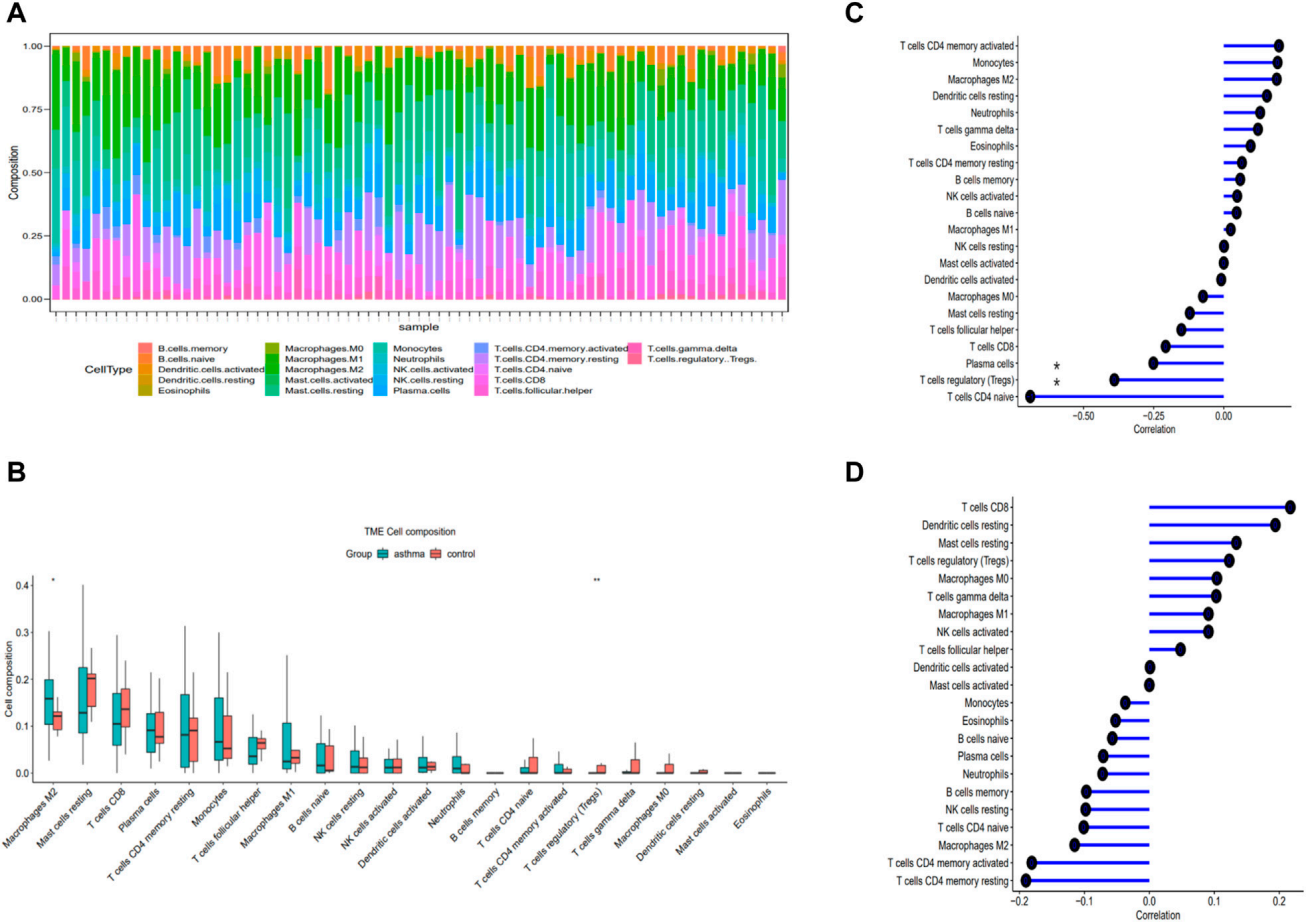
Evaluation of immune cell infiltration and correlational analysis. **(A)** Barplot shows the proportion of 22 types of immune cells in asthma. **(B)** The identification of differentially infiltrating immune cells **(C)** The correlation analysis of CISD1 and immune cells. **(D)** The correlation analysis of CAMKK2 and immune cells.

The top 5 immune cell subtypes with the highest infiltration proportion in the asthma group were macrophages M2, mast cells resting, T-cell CD8, plasma cells, and T-cell CD4 memory resting (Figure 4A). Compared with the healthy control group, the infiltration proportion of Regulatory T-cell (Tregs) (*p* < 0.05) decreased significantly in the asthma group, and macrophages M2 (*p* < 0.01) increased significantly in the asthma group (Figure 4B). In addition, we found a negative correlation between CISD1 and Tregs or plasma cells in Figure 4C (*p* < 0.05). In contrast, CAMKK2 was not involved in immune cell infiltration in Figure 4D (*p* > 0.05).

### 3.7 Validation

**FIGURE 5:**
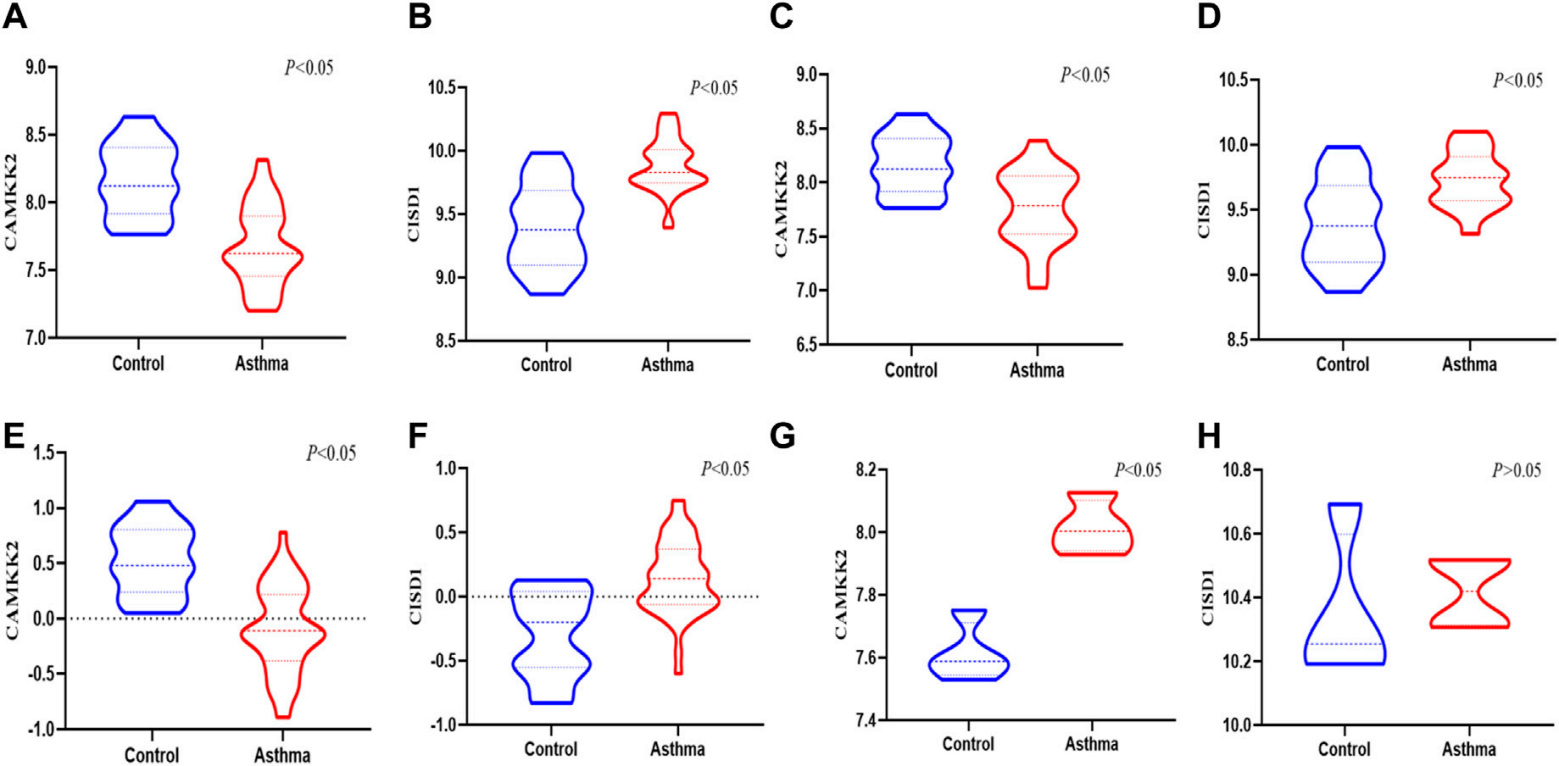
The validation of CAMKK2 and CISD1 in the GEO datasets **(A,B)** the expression of CAMKK2 and CISD1 in the severe asthma were verified in GSE 147878 (*p* < 0.05) **(C,D)** The expression of CAMKK2 and CISD1 in the mild/moderate asthma were verified in GSE 147878 (*p* < 0.05) **(E,F)** The expression of CAMKK2 and CISD1 in the severe asthma were verified in GSE 143303(*p*<0.05) **(G,H)** The expression of CAMKK2 and CISD1 were verified in GSE 27066 (*p* < 0.05)

CISD1 was significantly upregulated and CAMKK2 was downregulated in both mild to moderate asthma and severe asthma compared to healthy controls in the GSE147878 dataset (Figures 5A–D) (*p* < 0.05), which was consistent with the results of this study. Similar results were also obtained in the GSE143303 dataset, CAMKK2 was significantly downregulated in the asthma group, and CISD1 was significantly upregulated in the asthma group (Figures 5E, F) (*p* < 0.05). In addition, we found that CISD1 was also upregulated in the OVA group with no statistically significant difference (*p* > 0.05) in the GSE27066. Interestingly, CAMKK2 was also significantly upregulated in the OVA group (Figures 5G, H) (*p* < 0.05).

**FIGURE 6:**
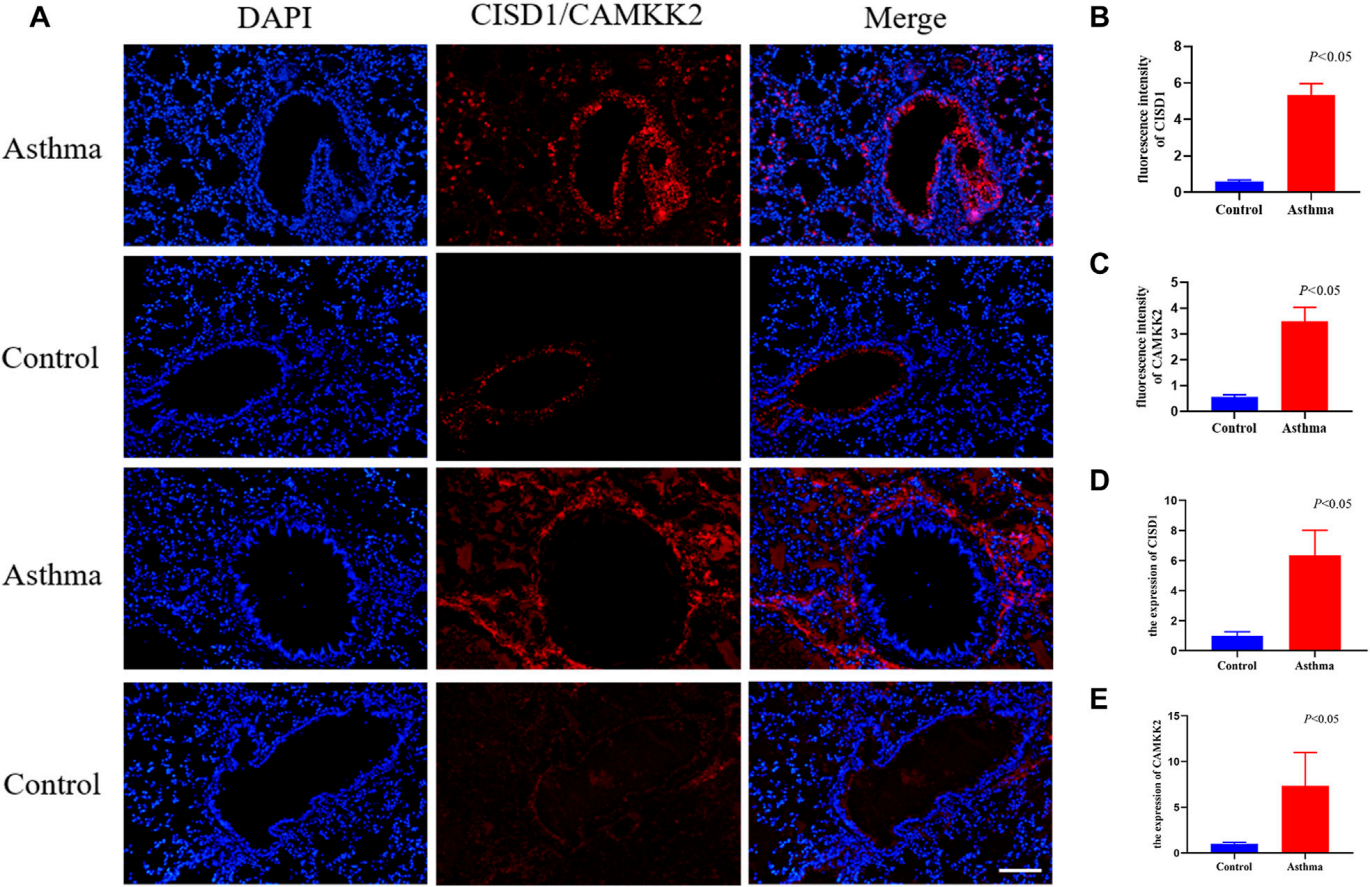
The result of validation by immunofluorescent staining and RT-qPCR in mouse models **(A–C)** immunofluorescent staining images of lung tissues and quantification of fluorescence intensity, Nucleus (blue), CISD1/CAMKK2 (red), scale bar, 500 um, (*n* = 4 per group) **(D–E)** The expression of CISD1 and CAMKK2 verified by RT-PCR in the OVA model (*n* = 4 per group).

IF staining was performed to verify the expression of CISD1 and CAMKK2 between the OVA group and the control group. CISD1 was highly expressed mainly in the airway epithelium of OVA mice, while CAMKK2 was widely expressed around the airway in Figure 6A. The results after fluorescence quantification showed that CISD1 and CAMKK2 were overexpressed in the OVA group compared to the control group in Figures 6B, C (*p* < 0.05). The RT-PCR results were also consistent with the staining results (Figures 6D, E) (*p* < 0.05).

## 4 Discussion

This study was the first to use WGCNA to investigate the role of genes associated with ferroptosis in asthma. We discovered that CAMKK2 and CISD1 were crucial ferroptosis suppressors in asthma. It was interesting that there was a negative correlation between the gene expression of CISD1 and Tregs, suggesting that CISD1 might be tied to the immunological microenvironment. This result suggested that ferroptosis might play important function in asthma.

Ferroptosis, a novel form of non-apoptotic cell death, was regulated by multiple cellular metabolic pathways, including redox homeostasis, iron handling, mitochondrial activity, and metabolism of amino acids, lipids, and sugars (Li and Li, 2020; Jiang et al., 2021). According to recent studies, various illnesses’ pathogenic processes and ferroptosis were related (Liang et al., 2019; Chen et al., 2021; Wu et al., 2021). Cell death-related disorders might benefit from ferroptosis therapy, according to certain research (Tang et al., 2021). Numerous studies conducted recently have discovered that ferroptosis was crucial to asthma. According to some studies, the malfunctioning of airway epithelial cells caused by ferroptosis might be the cause of asthma control loss. In contrast, inhibiting ferroptosis might reduce inflammation associated with asthma (Wenzel et al., 2017; Zeng et al., 2022). And Wu et al. (2020) have found that ferroptosis inducers could relieve allergic airway inflammation. It was still debatable whether ferroptosis and asthma were related. As we all know, asthma was characterized by the infiltration and activation of immune cells such as eosinophils, neutrophils, lymphocytes, and mast cells (Hammad and Lambrecht, 2021). The investigation of immune cells and ferroptosis together might result in a discussion of possible therapeutic applications.

CISD1, a mitochondrial protein mitoNEET was an iron-containing outer mitochondrial membrane protein with 13 kDa. Additionally, it was crucial for iron and reactive oxygen species (ROS) homeostasis detection and regulation (Geldenhuys et al., 2014; Lipper et al., 2019). The results of the enrichment analysis in this study also showed that CISD1 was mainly located in the cytoplasmic side of mitochondrial outer membrane, meanwhile, it was involved in the iron-sulfur cluster binding, 2 iron, and 2 sulfur cluster binding. These biological processes were highly associated with ferroptosis. However, no studies were conducted to explore the role CISD1 plays in asthma. In the present study, we found that CISD1 was significantly upregulated in the asthma group compared to healthy controls in asthmatic or OVA mice. Previous findings have consistently shown that CISD1 negatively regulated ferroptosis by protection against mitochondrial lipid peroxidation in Cancer (Yuan et al., 2016; Wang et al., 2021a).

Interestingly, we discovered a negative correlation between CISD1 and Tregs, pointing to the potential role of CISD1 in immune cell infiltration. Tregs were viable target against airway allergic inflammatory responses, and played an indispensable role in the maintenance of immune tolerance in asthma (Li et al., 2021; Zhang et al., 2022). By upregulating immunosuppressive molecules and suppressing genes, the Tregs fraction of CD4^+^ T-cell prevented the development of proinflammatory activities and reduced inflammation (Burzyn et al., 2013; van der Veeken et al., 2016). One intriguing study suggested that generating highly suppressive allergen-specific Tregs could alleviate the inflammatory and allergic aspects of asthma (Burzyn et al., 2013; Dembele et al., 2021). Tregs undoubtedly contributed significantly to asthma. A correlation between ferroptosis and immune infiltration has been found in cancer disease (Wang et al., 2021b; Hu et al., 2021). No studies, however, have found a connection between ferroptosis and immune infiltration in asthma. Ye et al. discovered in gliomas that CYP2E1 was engaged in lipid metabolism, ferroptosis, and associated to the tumor immune microenvironment, due to its significant link with Treg levels (Ye et al., 2021). Further investigation on the precise mechanisms governing ferroptosis and immune infiltration in asthma was anticipated in future studies.

CAMKK2 was a member of the serine/threonine-specific protein kinase family (Marcelo et al., 2016). It was a key regulator of glucose metabolism, insulin production, adipogenesis, and inflammation (Williams and Sankar, 2019). We found that CAMKK2 was downregulated in asthmatics and upregulated in animal models of asthma. The inconsistent results were mainly due to the 99% of the asthmatics in this study inhaled glucocorticoids (Shaw et al., 2015) and that glucocorticoids might downregulate CAMKK2. Our previous studies have also found that glucocorticoids downregulated several genes (Wang H. et al., 2022). Although this hypothesis has not been verified in other studies, we look forward to future studies to explore it. We, therefore, believe that CAMKK2 was upregulated in asthma, which in turn inhibited ferroptosis. CAMKK2 was considered to be an inhibitor of ferroptosis. Wang et al. showed that the suppression of CAMKK2 increased the efficacy of the ferroptosis inducer and inhibited the AMPK‒NRF2 pathway and promoted ferroptosis (Wang S. et al., 2022). In addition, we found that CAMKK2 was mainly involved in the CAMKK-AMPK signaling cascade, and the Adipocytokine signaling pathway, which was strongly correlated with the development of ferroptosis. Furthermore, our enrichment analysis revealed that CAMKK2 was involved in the autophagy of mitochondrion. Autophagy and ferroptosis were distinct patterns of cell death, and several studies have shown that CAMKK2 did activate mitochondrial autophagy (Lin et al., 2021; Xie et al., 2021), while a study has also found that the activation of autophagy protected cells from ferroptosis and the release of mitochondrial DNA (Zhao et al., 2020). We hypothesize that upregulated CISD1 might activate autophagy, whereas it would inhibit ferroptosis.

Our study was the first to find that upregulated CISD1 and CAMKK2 inhibited ferroptosis and played an important regulatory role in asthma. However, there were some weaknesses in this study. Firstly, we did not obtain consistent results for CAMKK2, which was downregulated in asthma patients and upregulated in the OVA mouse model. We speculated that the inconsistent results were mainly due to the use of inhaled glucocorticoids in asthma patients. However, there was no evidence that glucocorticoids downregulated CAMKK2, so future studies are urgently needed to investigate this problem. Furthermore, the asthma group in this study included patients with different severity of asthma (mild/moderate and severe), and severe asthma accounted for 70% of the patients, our results might only be an effect of severe asthma. Therefore, we verified the expression of CISD1 and CAMKK2 in mild/moderate asthma and severe asthma. We found CISD1 was significantly upregulated and CAMKK2 was downregulated in both mild to moderate asthma and severe asthma compared to healthy controls.

## 5 Conclusion

We discovered CAMKK2 and CISD1 were ferroptosis-related key genes for asthma patients, which could provide a reference for immunotherapies and targeted therapies.

Further investigation of the role and mechanism of the ferroptosis-related genes in the progression of asthma is still needed.

## Data Availability

The datasets presented in this study can be found in online repositories. The names of the repository/repositories and accession number(s) can be found in the article/Supplementary Material.

## Abbreviations

CAMKK2, Calcium/calmodulin-dependent protein kinase 2; ceRNA, competing endogenous; CISD1, CDGSH iron sulfur domain 1; FDR, false discovery rate; FEV1, forced expiratory volume in 1 s; GEO, Gene Expression Omnibus; GO, Gene Ontology; GS, gene significance; KEGG, Kyoto Encyclopedia of Genes and Genomes; MAD, median absolute deviation; MM, module membership; OCS, oral corticosteroid; PEF, peak expiratory flow; PPI, Protein-Protein Interaction network; RMA, robust multi-array average; RNAInter, RNA Interactome Database; ROS, reactive oxygen species; RT-qPCR, RNA real-time quantitative PCR; Tregs, Regulatory T-cell; WGCNA, Weighted Gene Co-expression analysis.
