# Supplementary material for: Ferroptosis-related genes are involved in asthma and regulate the immune microenvironment
Source: Front Pharmacol. 2023 Feb 10;14:1087557. doi: 10.3389/fphar.2023.1087557 (PMC9950254; doi:10.3389/fphar.2023.1087557)
Supplement: Supplementary file 1 [file DataSheet1.docx]

| Table S1: The characteristics of participants in the GSE147878 | | | | | |
| --- | --- | --- | --- | --- | --- |
| Characteristics | | HC(n=13) | mild/moderate asthma(n=18) | severe asthma (n=42) | *P* value |
| age |  | 45.31±12.82 | 60.94±14.75 | 57.21±12.05 | 0.309 |
| gender | male | 7(53.85%) | 3(16.67%) | 18(42.86%) | 0.072 |
|  | female | 6(46.15%) | 15(83.33%) | 24(57.14%） |  |
| OCS | no | 13(100.00%) | 18(100.00%) | 35(83.33%） | 0.057 |
|  | yes | 0(0.00%) | 0(0.00%) | 7(16.67%) |  |
| smoker | nonsmoker | 11(84.62%) | 16(88.89%) | 32(76.19%) | 0.482 |
|  | former smoker | 2(15.38%) | 2(11.11%) | 10(23.81%) |  |
| atopy | no | 12(92.31%) | 11(61.11%) | 18(42.86%) | 0.006* |
|  | yes | 1(7.69%) | 7(38.89%） | 24(57.14%) |  |
| prednisone | no | 13(100.00%) | 18(100.00%) | 36(85.71%) | 0.090 |
|  | yes | 0(0.00%) | 0(0.00%) | 6(14.29%) |  |
| **P*<0.05; OCS: oral corticosteroid; HC: health control | | | | | |

| Table S2: The characteristics of participants in the GSE143303 | | | |
| --- | --- | --- | --- |
| Characteristic | HC (n=13) | Severe asthma(n=47） | P value |
| Age(years), mean (SD) | 45 (13) | 59 (13) | 0.002* |
| Gender, Female n (%) | 6 (46) | 30 (64) | 0.250 |
| Atopy, n (%) | 1 (8) | 24 (51) | 0.004* |
| FEV1%predicted, mean (SD) | 101.4 (10.7) | 75.2 (21.5) | <0.001* |
| FVC %predicted, mean (SD) | 104.2 (9.9) | 91.8 (18.2) | 0.022* |
| FEV1/FVC, mean (SD) | 79.8 (3.9) | 66.0 (12.7) | <0.001* |
| Former smoker, n (%) | 2 (15) | 9 (19) | 0.756 |
| Pack years, median (Q1, Q3) | 5 (5, 5) | 10 (5, 15) | 0.275 |
| Total cell count x106/mL, median (Q1, Q3) | 0.1 (0.1, 0.2) | 0.2 (0.1, 0.6) | 0.170 |
| Viability, median (Q1, Q3) | 75 (68, 76) | 80 (57, 91) | 0.440 |
| Neutrophils %, median (Q1, Q3) | 40.3 (15.3, 62.8) | 56.0 (24.5, 81.5) | 0.193 |
| Eosinophils %, median (Q1, Q3) | 0.5 (0.3, 1) | 2 (0.8, 9.8) | <0.001* |
| Macrophages %, median (Q1, Q3) | 33 (20.8, 43.8) | 23.8 (11.5, 40.8) | 0.149 |
| Lymphocytes %, median (Q1, Q3) | 1.5 (0.5, 2) | 0.5 (0, 2.3) | 0.183 |
| Epithelial cells %, median (Q1, Q3) | 14.5 (7.5, 23.3) | 3.5 (0.8, 15.8) | 0.010* |
| Squamous cells %, median (Q1, Q3) | 2 (1, 2.4) | 1.5 (0, 3.2) | 0.342 |
| **P*<0.05; HC: health control | | | |
